# Success and limitations in adaptation of Fast-TrACC tissue culture-independent transformation in coffee, cotton, and tree tobacco

**DOI:** 10.1371/journal.pone.0318324

**Published:** 2025-05-15

**Authors:** Boaz Negin, Laura Daniela Martin-Lesmes, Georg Jander

**Affiliations:** 1 Boyce Thompson Institute, Ithaca, NY, United States of America; 2 Universidad de los Andes, Bogotá, Colombia; United States Department of Agriculture, UNITED STATES OF AMERICA

## Abstract

Plant transformation is a critical process for generating transgenic and genome-edited plants for use in research and agriculture. For most plant species, this process has traditionally involved genomic insertion of DNA in tissue culture and regeneration of transformed plants through hormonal induction. Recently, methods for plant transformation in a tissue culture-independent manner, through the expression of growth regulators, have been published. We attempted to adapt this promising approach to three woody species, coffee (*Coffea arabica*), cotton (*Gossypium hirsutum*), and tree tobacco (*Nicotiana glauca*), using a combination of *Agrobacterium* strains, plasmid systems, and different promoters driving the expression of *ZmWUS2* and *AtIPT*, which were originally adapted for this purpose in *Nicotiana benthamiana*. We found that tree tobacco was amenable to tissue culture-independent transformation but had difficulty developing transgenic seeds. Coffee was not receptive to this transformation method, and cotton was amenable to callus formation but did not exhibit gene insertions in the newly-formed shoots. These limitations are partially technical, such as maize *WUS* not affecting coffee similarly to other plants, but are in part fundamental setbacks in the use of growth regulators to drive tissue culture-independent transformation. We suggest how these drawbacks can be overcome in the future through the use of inducible or tissue-specific promoters and other means.

## Introduction

Genetically engineered plants have been utilized in research and agriculture for decades. Currently, more than 90% of US corn, soybeans, and upland cotton are produced using genetically engineered varieties [1], and genetic engineering for plant research has become a routine process, especially in the case of model organisms. Furthermore, the CRISPR revolution [2–4], which enables generating precise knock-out mutations of almost any desired gene, also requires a stage of genetic engineering. However, in this respect, not all plants are created equal. Some plants have an established floral dip transformation system [5–7], which does not require passage through tissue culture, but merely dipping flowers in an *Agrobacterium* solution and then collecting transformed seeds. For other plant species, there are simple, straightforward transformation systems involving tissue culture [8–10]. In this case, plants are grown in sterile conditions, are infected with *Agrobacterium*, or more rarely, have DNA inserted into cells using a gene gun [11], and then are passed through several stages in different media until a whole plant is regenerated from a single transformed cell. Regeneration efficiency is often a major issue in these methods, and there are plants that can be genetically engineered, but the low efficiency of regeneration makes this a slow and inefficient process [12]. Finally, there are plants, including most wild species, for which robust transformation systems remain to be developed.

The difference between studying a plant that has a well-developed floral dip transformation method and a plant that has no simple transformation system is immense. The questions we can ask, and the resources we will expend to answer them change completely between the two. Furthermore, although many processes can be studied in model species, there are entire fields in plant science, for instance specialized metabolism, that can be genus or family-specific and thus impossible to study in an established model system [13].

For these reasons, attempts to simplify plant transformation protocols and approaches are continually being developed. In recent years, tissue culture-independent transformation has been successfully developed by several labs. These methods include dipping cuttings in *Agrobacterium rhizogenes* and regenerating plants from transgenic roots [14,15], injecting *Agrobacterium* into pollen tubes of peanut plants [16], and a shoot apical meristem cell-mediated transformation system in cotton [17] and *Corchorus* [18]. Regenerating from transgenic roots can work simply for some plants, but many others will not regenerate from this single tissue without further intervention. Meristem direct-delivery methods tend to be very species-specific and will not work for most plants.

The recently developed Fast-TrACC method [19,20], which involves the use of growth regulators to induce *de novo* formation of shoots from which transgenic seeds can be collected, may provide a more universal method for tissue culture-independent plant transformation. In the Fast-TrACC approach, two growth regulators – a *WUSCHEL* (*WUS*) gene from maize and a cytokinin biosynthesis gene from *Agrobacterium tumefaciens* (*IPT*) are expressed together, or co-infiltrated with a gene of interest. The expression of the growth regulators leads to formation of ectopic shoots, from which transgenic seeds can be collected. Although the Fast-TrACC transformation method was originally developed with *Nicotiana benthamiana*, the genetic distance from a maize gene (*ZmWUS*) and an *Agrobacterium* gene (*AtIPT*) to *N. benthamiana*, is no closer than to other eudicots, suggesting that the two growth regulators may have broad applications in plant transformation.

In most cases, tissue culture-independent transformation requires a robust reporter system. Whereas tissue culture-mediated transformation routinely uses antibiotic resistance genes for transgene selection, this cannot be used to select transgenic plant organs in the T0 generation, if only because trying to select a transgenic branch on a wildtype plant would inevitably lead to the entire plant dying. In the examples mentioned above, different reporters were used, including GUS staining, GFP, bleaching following mutations in the phytoene desaturase (*PDS*) gene, and betalain production. This last method, was made possible by the recent discovery of the betalain biosynthesis pathway [21] and the engineering of a “betalain reporter”, which consists of three betalain biosynthetic genes that are expressed in a single open reading frame and are cleaved post-translationally by self-cleaving 2A peptides [22]. This reporter has several advantages over others that have been used previously – it is visible to the naked eye, has a stable product, does not require a gene-specific stage of genome editing, and is transferable to different plant species without further adaptation.

In the current study, we focused on three woody species, tree tobacco (*Nicotiana glauca*), cotton (*Gossypium hirsutum*), and coffee (*Coffea arabica*), in an attempt to develop tissue culture-independent transformation systems for the three. Tree tobacco was chosen for its genetic similarity to *N. benthamiana*, whereas cotton and coffee were selected due to their agricultural importance and the inefficiency of the existing transformation protocols. Developing a tissue culture-independent transformation system for these species would accelerate and simplify generation of transgenics and mutants, making molecular research in them accessible and relatively simple for many research groups, and in the long run also aid in breading of the agriculturally important coffee and cotton.

## Materials and methods

### Plant material and growth conditions

*Nicotiana benthamiana* plants were grown in growth rooms at 23°C in Cornell Mix [56% peat moss, 35% vermiculite, 4% lime, 4% Osmocote slow-release fertilizer (Scotts, Marysville, OH), and 1% Unimix (Scotts, Marysville, OH)] with a 16:8 light:dark cycle. *Nicotiana glauca* plants were grown using seeds that originated from the Weizmann Institute of Science, Rehovot, Israel, and were grown in conditions similar to *N. benthamiana* while young. Larger plants were transferred to a temperature-controlled greenhouse (26-28°C), with supplemental light added through high pressure sodium lamps when the ambient light intensity dropped below 350 μE. Seeds from six cotton (*Gossypium hirsutum*) cultivars, upland cotton, UGA230, Pronto (PI 529594, SA 1540), Coker310 (PI 529249, SA 1184), Tipo Chaco (PI 528557, SA 0159) and Delta Pine 16 (PI 520251) were provided by Andrew Nelson (Boyce Thompson Institute). Cotton was grown in a growth room under the same conditions as the *N. benthamiana* plants. Coffee (*Coffea arabica*) fruit were provided by Jim Giovannoni (USDA-ARS) and were germinated and grown using the same conditions as *N. benthamiana* plants.

### *Agrobacterium* strains

*Agrobacterium* strains used in this study were *Agrobacterium tumefaciens* strains GV3101, LBA4404, and EHA 105, and the *Agrobacterium rhizogenes* strain MSU440. All strains were kept as glycerol stocks prior to growth in LB medium.

### Goldengate cloning

The original Fast-TrACC plasmids [20] were obtained from Addgene (www.addgene.org). These were Goldengate (GG) [23] plasmids using the A-B-C′-D module, and included pMOD_A0101 (Plasmid #90998) with *Cas9* driven by a *35S* promoter, pMOD_A3001 (Plasmid #91042) containing *35S::GFP*, pMOD_B2303 (Plasmid #91068) encoding a cassette for gRNA expression driven by a yellow leaf curl virus (YLCV) promoter and including tRNA spacers for gRNA cleavage following transcription, pMM100 (Plasmid #127225) – a B module plasmid encoding one gRNA following a *U6* promoter and targeting *N. benthamiana PDS*, pMOD_B0000 (Plasmid #91058) – an empty module B plasmid, pMOD_C’5014 (Plasmid #127219) containing *pNOS::ZmWUS2*, pMOD_D7101 (#127229) encoding a *35S::AtIPT*, pTRANS_221 (#91115) – a binary vector to which the different pMODS were inserted.

The four A-B-C’-D modules were inserted to pTRANS_221 using the Goldengate cloning technique [23]. Using this method, two binary plasmids were generated in pTRANS_221. The first was GFP + *NOS::ZmWUS* + *35S::AtIPT* and is referred to as “Goldengate-overexpression” or “GG-OE”. The second was *35S::Cas9* + *U6::PDS-gRNA +NOS::ZmWUS + 35S::AtIPT* referred to as “GG-Cas9”.

### Goldenbraid cloning

In addition to the Goldengate system, we adapted the Fast-TrACC method plasmids to the Goldenbraid (GB) cloning system [24] (plasmid maps and Goldenbraid cloning progression are presented in S1-[S10_Fig]S10 Figs). Initially, we domesticated genes and promoters from the Goldengate plasmids and inserted the domestication products into PUPD2 plasmids (Plasmid #68161; for primers used for domestication of Goldenbraid parts see S1 Table, for the construction workflow see S1 Fig). PUPD-*pNOS* (Plasmid #68166), PUPD2 *WUS* and PUPD *tNOS* (Plasmid #68188) were merged into an α1 plasmid (Plasmid #68228; S2 Fig), whereas PUPD2-*35S*, PUPD2-*IPT* and PUPD-*T35S* (plasmid #68187) were merged to an α2 plasmid (Plasmid #68229; S3 Fig). In parallel, the RUBY reporter, consisting of three betalain biosynthetic genes *CYP76AD1*, *l-DOPA 4,5-dioxygenase* (*DODA*), and *glucosyltransferase* [22], was domesticated into PUPD2. Several promoters were domesticated into PUPD2 as well, to examine which ones would lead to the strongest betalain production when regulating RUBY. The domesticated promoters included the *YLCV* promoter, amplified from pMOD_b2303, the *PPDK* promoter, amplified from a pFGC-pcoCas9 plasmid (Plasmid #52256), an enhanced *35S* promoter, amplified from a pTRANS_210D plasmid (Plasmid #91109) and the *35S* promoter, *NOS* promoter, and *UB10* promoters from the GB kit (Plasmids #68163, 68166, and 68174, respectively). These were inserted alongside RUBY into α1 plasmids, transferred to *Agrobacterium* strain GV3101, and infiltrated into *N. benthamiana* leaves. Following several such infiltrations, we assessed the betalain production intensity as NOS<35S<UB10<YLCV=e35S=PPDK. We chose to use the *YLCV* promoter, since this promoter sequence does not contain elements from the *35S* promoter, which regulates other genes in the final constructs. An α2_*YLCV::RUBY* plasmid was cloned (S6 Fig), and transferred to an Ω1 plasmid (Plasmid #68238) using an α1 stuffer plasmid (#68232; S9 Fig). In parallel, the α1_*NOS::WUS* and α2_*35S::IPT* plasmids were fused into an Ω2 plasmid (Plasmid #68235; S7 Fig). The Ω1_*YLCV::RUBY* and Ω2_*NOS::WUS + 35S::IPT* were then fused into an α1 plasmid (α1_*YLCV::RUBY+NOS::WUS+35S::IPT*; [S10_Fig]S10 Fig) referred to throughout the manuscript as “GB_*NOS::WUS”*. Similar to this process, the PUPD2_*e35S* and PUPD2_*WUS* plasmids were connected into an α1 plasmid (S4 Fig) and PUPD-*AtUB10* (Plasmid #68174) was connected with PUPD2::*IPT* and PUPD2-*SlUB10* terminator in an α2 plasmid (S5 Fig). After a similar process, a final α1_*YLCV::RUBY* + *e35S::WUS* + *UB10::IPT* plasmid was formed (S11 Fig), referred to throughout the text as “GB-*e35S::WUS*”. For a chart describing the plasmids delivered in different bacteria to the four plant species, see S12 Fig.

### Insertion of plasmids to *Agrobacterium* strains and agroinfiltration of *N. benthamiana* leaves

Finally, the binary plasmids were inserted to four *Agrobacterium* strains, using electroporation for GV3101, EHA105, and MSU440 and heat shock for LBA4404. GV3101 was used for agroinfiltration of *N. benthamiana* leaves when examining the different RUBY plasmids. For these infiltrations, the bacteria were shaken overnight in a 4 ml culture vile at 28°C, after which a subculture was made in 30 ml LB and shaken similarly. The culture was then centrifuged (4,000 g, 10 min), resuspended in infiltration buffer (10mM MgCl_2_,10 mM 2-(N-morpholino)ethanesulfonic acid (MES) pH 5.6, and 200 μM acetosyringone (AS; Sigma Aldrich , St. Louis, MO), diluted in this buffer to an OD600 of 0.4, shaken at room temperature for 2-3 h, and injected to leaves using a needleless 1 ml syringe [25].

### Plant infiltration

Plant injection to induce tissue culture-independent transformation followed the protocol published in [20]. In short, bacteria were shaken in LB to which 20 μM acetosyringone was added, cultures were diluted to an OD600 of 0.2, and injections were performed using an insulin injection needle.

**Fig 1:**
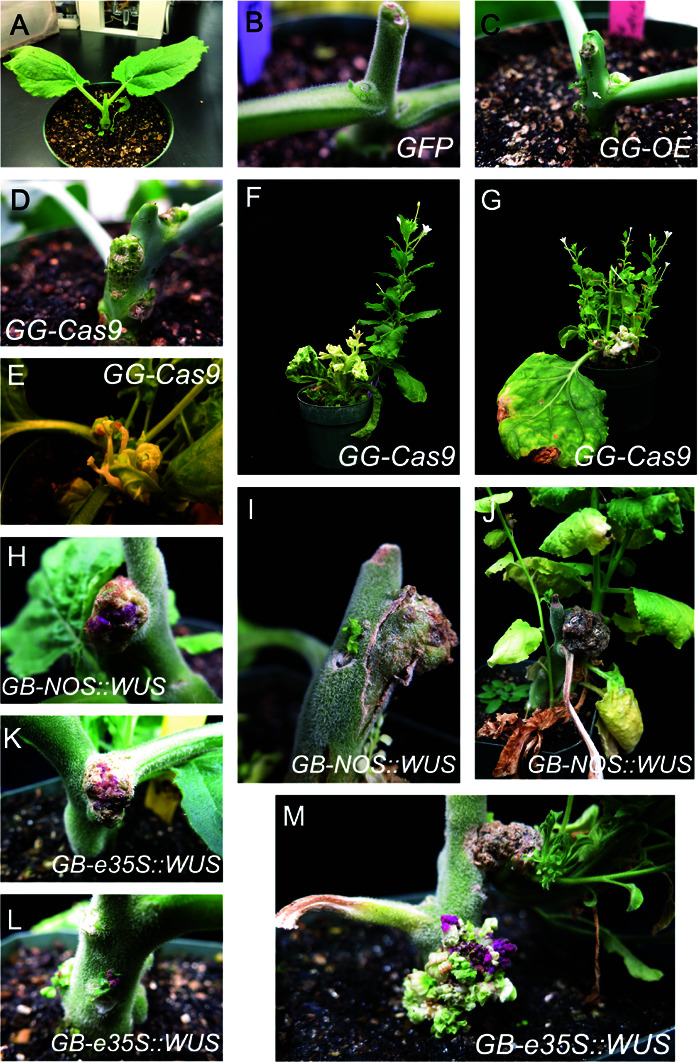
Tissue culture-independent transformation of Nicotiana benthamiana. A: An *N. benthamiana* plant whose leaves and apical meristem were removed prior to bacterial injection. B: A control plant, injected with an empty vector shows no ectopic shoot formation. C: A plant injected with the GG-OE plasmid shows ectopic shoot formation. D: A plant injected with the GG-Cas9 plasmid shows similar ectopic shoot formation. E-G: Plants injected with the GG-Cas9 plasmid targeting the *PDS* gene form deformed shoot like bleached structures. H-J: Plants injected with the GB-*NOS::WUS* plasmid, show callus formation, in one case (H) with pink betalain coloring. K-M: Plants injected with the GB-*e35S::WUS* plasmid show pigmented callus (K), pigmented shoot L: and callus with multiple pigmented shoots emerging from it (M).

**Fig 2 pone.0318324.g002:**
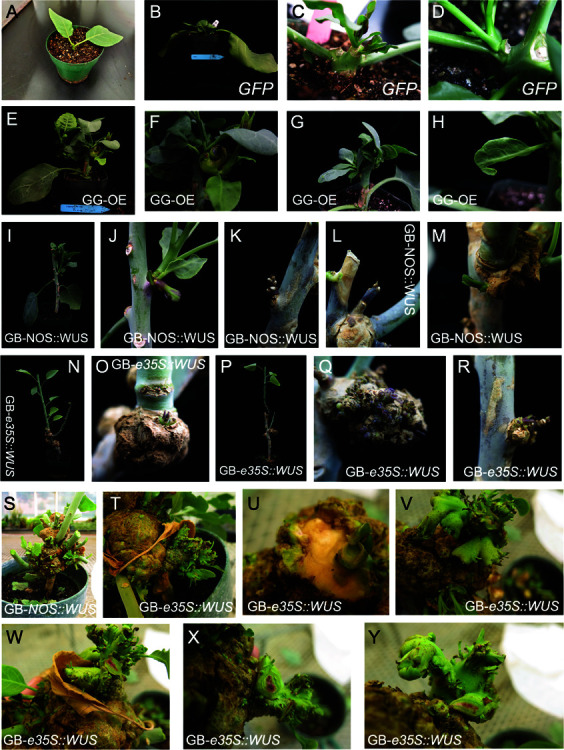
Tissue culture-independent transformation of tree tobacco (Nicotiana glauca). A: A tree tobacco plant whose leaves and apical meristem were removed prior to bacterial injection. B-D: Control plants, injected with an empty vector show no ectopic shoot formation, but develop new shoots from axillary buds. E-H: Plants injected with the GG-OE plasmid show denser shoots (E,G), deformed shoot-like structures (F) and deformed leaves (H). I-M: Plants injected with the GB-*NOS::WUS* plasmid show formation of purple deformed leaves (J), purple, dense shoots (K) and calli from which either purple (L) or green (M) shoots grow. N-R: Plants injected with the GB-*e35S::WUS* plasmid show large calli formation from which pigmented shoots develop. One of the calli shows the development of many (over 10) pigmented shoots (Q). (S-Y) Tree tobacco plants injected with both GB constructs and transferred to a greenhouse show large callus growth from which many shoots and shoot-like structure proliferated. When these shoot-like structures were cut W-Y: pink pigmentation can be seen in the center of the shoots.

**Fig 3:**
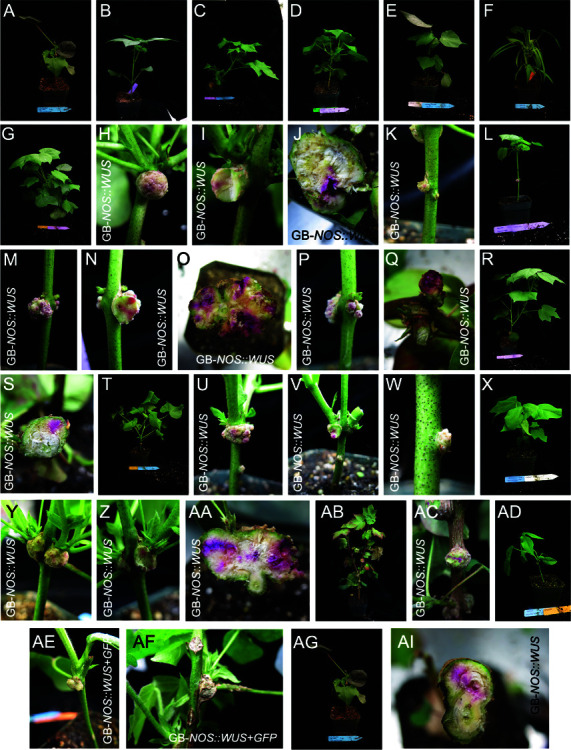
Tissue culture-independent transformation of cotton (Gossypium hirsutum). A-F: The six cotton cultivars examined in this study. A: Upland cotton. B: UGA230. C: Delta Pine 16. D: Tipo Chaco. E: Coker 310. F: Pronto. G-S: UGA230 cotton injected with the GB-*NOS::WUS* in *Agrobacterium* strain EHA105 (G-K), MSU440 (L-Q) and GV3101 (R-S). All calli display pink pigmentation both when whole and following cutting. T-W: Delta Pine 16 cotton injected with the GB-*NOS::WUS* construct in *Agrobacterium* strain MSU440, showing pigmented callus formation. X-AC: Coker 310 cotton injected with the GB-*NOS::WUS* plasmid in *Agrobacterium* strain LBA4404, showing pigmented callus formation. AD-AF: Pronto cotton injected with GB-*NOS::WUS*+*GFP* in *Agrobacterium* strain LBA4404 shows unpigmented callus formation. AG-AI: Upland cotton injected with the GB-*NOS::WUS* plasmid in *Agrobacterium* strain LBA4404 shows pigmented callus formation.

**Fig 4 pone.0318324.g004:**
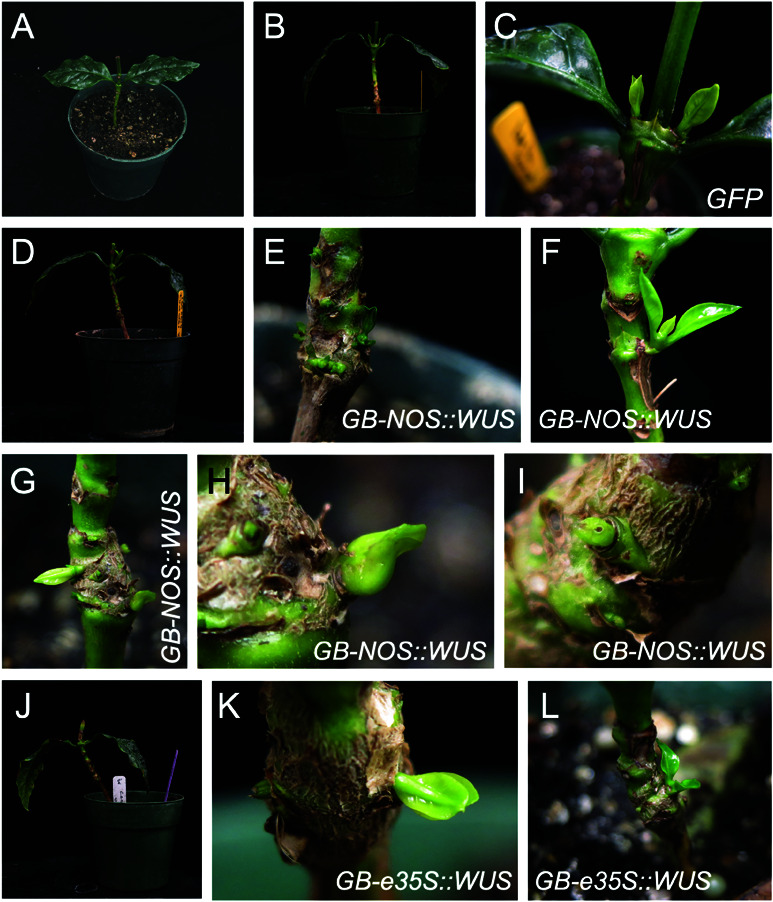
Tissue culture-independent transformation of coffee (Coffea arabica). A: A coffee plant whose leaves and apical meristem were removed prior to bacterial injection. B-C: A coffee plant injected with *GFP* in *Agrobacterium* strain LBA4404 showed normal shoot development from lateral buds. D-I: Coffee plants injected with the GB-*NOS::WUS* plasmid in *Agrobacterium* strain LBA4404 show dense shoot proliferation in regions which are not adjacent to lateral buds. J-L: Coffee plants injected with the GB-*e35S::WUS* plasmid in *Agrobacterium* strain LBA4404 show shoot development from non-lateral bud locations.

The four plant species used in this study were infiltrated in the seedling stage. The *N. benthamiana* plants were injected following the beginning of branching, whereas the others were injected when plants had approximately 4-6 true leaves. At this stage, all leaves, buds and the shoot apical meristem were removed and only two “helper leaves” were left (Figs 1A,2A,3A, and 4A). Bacterial cultures were injected at the stem cut site, leaf cutting sites, buds and in tree tobacco, the needle was inserted under the stem epidermis and bacterial solution was injected along the stem. Plants were then returned to the growth room where new growth was removed for approximately 20 days, after which new growth was not removed. Once shoots suspected to be transgenic were identified, other shoots were occasionally pruned to prevent the plant parts that were unhindered by the *WUS* and *IPT* growth regulators from taking over.

### DNA extraction

DNA was extracted from leaves that were suspected to be transformed based on morphology, or originating from calli. In addition, presumed non-transformed leaves and some callus tissues were sampled. Plant tissue was ground using steel beads and a tissue homogenizer (GenoGrinder 1600 MiniG homogenizer, SPEX SamplePrep, Metuchen NJ, USA) in a buffer consisting of 200 mM Tris-HCl pH=7.5, 250 mM NaCl, and 25 mM ethylenediaminetetraacetic acid (EDTA), to which 0.25% sodium dodecyl sulfate (SDS) was added following grinding. Centrifugation was used to remove cell debris, isopropanol was added at a volume equal to the supernatant and left for 10 min at room temperature to precipitate DNA followed by centrifugation in a Beckman GS-6R centrifuge (Beckman Coulter, Brea, CA) for 35 min at 1,700g. The pellet was washed with 70% ethanol, and DNA was resuspended in double distilled water. For difficult tissues, such as coffee leaves, which had soluble components that inhibited PCR reactions, a NucleoSpin^®^ Plant II DNA extraction kit (Takara Bio, Kusatsu, Shiga, Japan) was used, according to the manufacturer’s instructions.

### PCR reactions for validation of construct presence

PCR reactions were performed using GoTaq Green Master Mix (Promega, Madison, WI) or Q5 *Taq* polymerase (New England Biolabs, Ipswich, MA) according to the manufacturers’ instructions. The targeted genes were either from the RUBY construct, or the *WUS* gene (see S1 Table for primers used).

## Results

### Transformation of *Nicotiana benthamiana*

To validate that we could reproduce successful tissue culture-independent transformation of *N. benthamiana*, we applied the Fast-TrACC protocol developed by Cody *et al*. [20], using the original plasmids used in this study. In addition, we adapted the genes and promoters to the Goldenbraid cloning system, which allows a more modular construction of promoter - gene combinations and the addition of additional genes, such as reporter genes. As a reporter, we utilized the RUBY construct [22], which drives the production of betalains through the expression of three biosynthetic genes.

Altogether, we transformed *N. benthamiana* with four constructs using *Agrobacterium* strain GV3101: (i) a Goldengate plasmid system containing *NOS::WUS*, *35S::IPT*, and *GFP* (GG-OE), (ii) a Goldengate system plasmid with *NOS::WUS*, *35S::IPT*, *Cas9*, and a guide targeting the *N. benthamiana PDS* gene (GG-Cas9), (iii) a Goldenbraid system plasmid to which the original genes and promoters used in [20]. were domesticated – *NOS::WUS* and *35S::IPT* and the RUBY reporter driven by a *YLCV* promoter was added (GB-*NOS::WUS*; [S10_Fig]S10 Fig), and (iv) a Goldenbraid system plasmid in which the *WUS* and *IPT* genes were driven by an enhanced *35S* promoter (*e35S*) and a *UB10* promoter respectively, and to which *YLCV::RUBY* was also fused (GB-*e35S::WUS*; S11 Fig).

All buds and the apical meristem were removed from *N. benthamiana* plants (Fig 1A). Approximately three weeks after injection with *Agrobacterium* containing the GG-OE plasmid, during which new growth was repeatedly removed, new shoot growth could be seen emerging from regions of the plant where it does not normally develop (Fig 1C), such as from lateral buds (Fig 1B). However, this new growth had arrested development and was soon overwhelmed by the plants’ normal growth. The GG-PDS construct first led to similar ectopic shoot development (Fig 1D), followed by development of shoot-like structures, which were bleached due to induced mutations in the *PDS* gene (Fig 1E-G). Similar to the GG-OE plants, the bleached shoots had severely retarded development and did not produce viable flowers (Fig 1F-G). The plants transformed with the GB plasmids showed both callus and ectopic shoot formation (Fig 1H-M), with the GG-*NOS::WUS* construct driving formation of calli, ectopic shoots (Fig 1I-J), and betalain-producing calli (Fig 1H). The GB-*e35S::WUS* construct drove formation of betalain-producing calli (Fig 1K), shoots (Fig 1L; as seen by the presence of trichomes), and a combination of large calli from which multiple betalain-producing shoots emerged (Fig 1M). Despite these promising results showing formation of reporter-expressing shoots, none of these and other shoot-like structures developed into a grown shoot, and no transgenic seeds could be collected.

### Transformation of *Nicotiana glauca*

Since the original method we were attempting to adapt was developed for *N. benthamiana*, we first attempted to adapt it to a woody relative, tree tobacco. We transformed these plants with four different plasmid/promoter combinations: (i) The original GG-OE plasmid, (ii) GB-*NOS::WUS*, (iii) the stronger expression GB-*e35S::WUS*, and (iv) a GFP control without growth regulators.

Similar to *N. benthamiana*, tree tobacco plants transformed with a construct not containing the growth regulators developed new shoots from the regions of the lateral buds, although this development was much faster and more vigorous than with *N. benthamiana* (Fig 2B-[Fig pone.0318324.g002]D). Plants transformed with the GG-OE plasmid showed development of deformed, denser shoots with deformed shoot-like structures (Fig 2E-[Fig pone.0318324.g002]H), indicating that these were expressing the growth regulators. Plants into which the GB-*NOS::WUS* plasmid was transformed developed purple deformed leaf-like structures (Fig 2H-[Fig pone.0318324.g002]I) and calli from which purple and green shoots developed. When the plants were transformed with the GB-*e35S::WUS* construct, large calli were formed from which purple shoots and shoot-like structures emerged. Due to the large size of tree tobacco, plants were transferred to a greenhouse where we tried to achieve seed production from transgenic shoots. In the greenhouse, calli continued developing and many shoot-like structures were formed, mainly in GB-*e35S::WUS* transformed plants (Fig 2S-[Fig pone.0318324.g002]Y). When these shoot-like structures were cut to examine whether they produce betalains, a purple core could be seen (Fig 2W-[Fig pone.0318324.g002]Y), indicating that the structures were indeed transgenic.

Despite this successful transformation of the shoots, when progeny of shoots that originated from calli were grown, these had a wildtype phenotype, with neither purple coloring nor growth deformities. PCR reactions targeting the *WUS* and *RUBY* genes were not able to amplify them, indicating that these progeny were not transgenic.

### Transformation of cotton

Being an agriculturally cultivated species, there are many cotton cultivars, with potentially different sensitivity to transformation. We therefore attempted tissue culture-independent transformation using six cotton cultivars (upland cotton, UGA230, Pronto, Coker 310, Tipo Chaco, and Delta Pine 16; Fig 3A-F) and four *Agrobacterium* strains (GV3101, EHA105, LBA4404 and MSU440). The *Agrobacterium* strains were transformed with the two GB plasmids and injected to cotton seedlings. The UGA230 cultivar transformed with *Agrobacterium* strain EHA105 containing the GB-*NOS::WUS* construct led to the formation of calli with pink coloring. When these calli were cut, the coloring could be seen to be produced in the different callus layers. (Fig 3G-K). UGA230 was similarly susceptible to *Agrobacterium* strain MSU440 containing the same plasmid (Fig 3M-R), and to a lesser extent to the GV3101 strain, but nonetheless produced a callus with pink coloration (Fig 3S-T). Delta Pine16 plants transformed with MSU440 expressing the GB-*NOS::WUS* construct similarly generated calli with pink coloring (Fig 3T-W), as did plants from the Coker cultivar transformed with LBA4404 and expressing the same construct (Fig 3X-AC). Plants from the Pronto cultivar transformed with LBA4404 expressing GB-*NOS::WUS* lacking the RUBY reporter, and co-infiltrated with a *GFP* expression plasmid formed calli, although *GFP* expression in this was difficult to observe due to autofluorescence. (Fig 3AD-AF). Finally upland cotton transformed with LBA4404 carrying the GB-*NOS::WUS* plasmid generated calli with pink coloring (Fig 3AG-AI).

Despite this extensive formation of betalain-producing calli, demonstrating the expression of the reporter genes, no betalain-colored leaves were produced, and the presence of the transgenes could only be confirmed by PCR in the calli themselves and not in leaves (S13 Fig), even when shoots seemed to be originating directly from the calli.

### Transformation of coffee

Accelerating coffee transformation and making it accessible to a greater number of research groups can greatly improve both research and breeding of this economically important plant. We therefore examined the suitability of tissue culture-independent transformation of coffee. In a preliminary trial, we observed that *Agrobacterium* strain LBA4404 seemed to generate the best results, in terms of new shoot proliferation. Due to the long time coffee requires for germination and initial growth, sometimes reaching six months before plants can be used, we chose to focus our efforts on this strain, using both the GB-*NOS::WUS* and GB-*e35S::WUS* constructs. Whereas control, *GFP* transformed plants seemed to develop new shoots mainly from axillary buds (Fig 4B-C), plants transformed with both constructs containing growth regulators (Fig 4C-[Fig pone.0318324.g004]K), but especially those transformed with GB-*NOS::WUS* (Fig 4D-I), formed shoots from non-axillary bud regions. Furthermore, these shoots tended to be more densely clustered than the control plants (Fig 4E and 4G). However, no pink coloring could be seen in the shoots, no calli were formed, and the presence of the transgenes could not be confirmed by PCR.

### Additional approaches for tissue culture-independent transformation

The Arabidopsis *Wuschel* gene can drive organogenesis only when expressed in pluripotent tissues [26]. These exist naturally throughout plants, in buds, apical growth meristems, and, in woody species, also the cambium. Another place where pluripotent plant tissue is formed, is in natural callus formation, following wounding. Hence, we attempted to utilize this natural dedifferentiation following wounding in more mature plants where a woodier stem had grown, by wounding the stems and injecting *Agrobacterium* into wound sites or by dipping cuttings in *Agrobacterium*.

**Fig 5 pone.0318324.g005:**
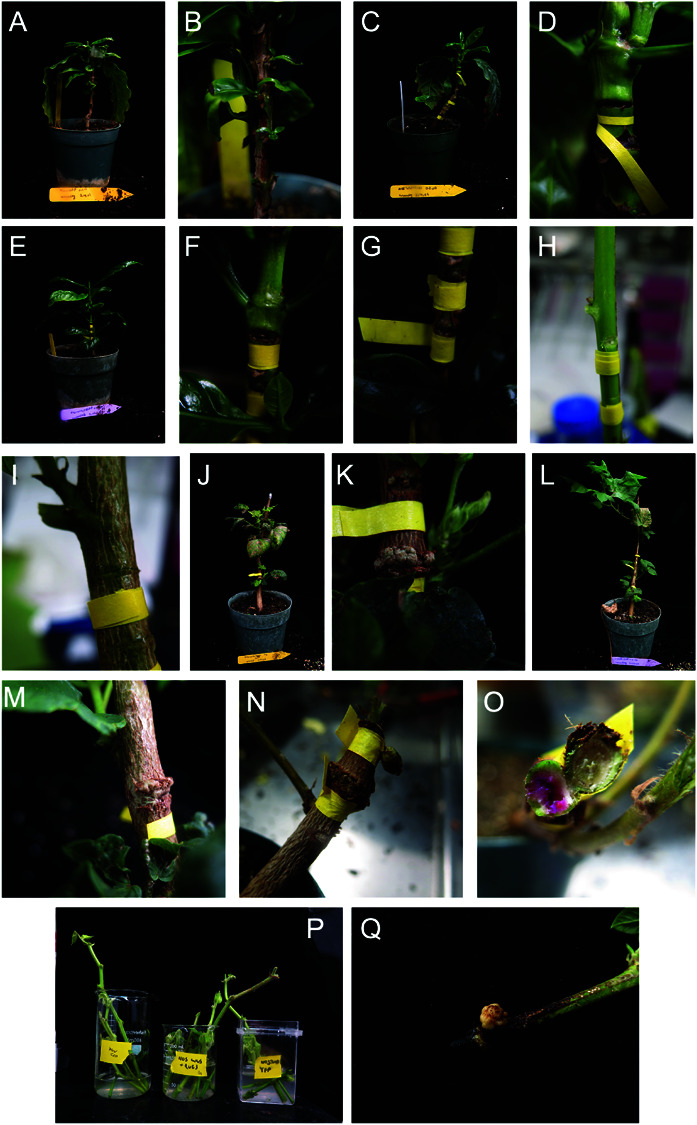
Additional approaches to induce tissue culture-independent transformation. A-O: injection of *Agrobacterium* to wound sites in stems. P-Q: Dipping cuttings in *Agrobacterium* containing growth regulator plasmids. A-G: Injected wound site in coffee plants. No shoot or callus formation can be seen in these. H-O: Injected wound sites in young (H) and more mature (I) cotton plants. J-M: Beginning of calli formation around wound sites. N-O: Formation of a betalain producing callus from an injected wound site. P: Coffee and cotton cuttings in three *Agrobacterium* strain LBA4404 suspensions, including constructs – *YLCV::RUBY* (control vector), GB-*NOS::WUS*, and GB-*NOS::WUS*+*YFP*. (Q) A cotton cutting dipped in GB-*NOS::WUS*+*YFP,* which developed a callus near the cut site. .

We attempted this for both cotton and coffee (Fig 5), generating deep wounds at three locations along the stem (Fig 5H and 5I). Cuttings were inserted into the *Agrobacterium* strain LBA4404 with three constructs: (i) *YLCV::RUBY* (control), (ii) GB-*NOS::WUS*, and (iii) GB-*NOS::WUS* + *YFP* (Fig 5P). Coffee plants targeted in this way did not respond to the transformation, with wounding sites remaining without shoot or callus formation when injected with both GB-*NOS::WUS* and GB-*e35S::WUS* constructs (Fig 5A-[Fig pone.0318324.g005]G). Younger cotton plants, which were treated similarly, also did not respond. However, more mature cotton plants developed calli from the wound sites (Fig 5J-[Fig pone.0318324.g005]M), and almost five months after the initial wounding and infiltration, a pink callus was excised from one of the transformed plants (Fig 5N-[Fig pone.0318324.g005]O). The cotton cuttings had an even smaller success rate, although one cutting, which had been dipped in the GB-*NOS::WUS* +*YFP* construct, did develop a callus at the bottom of the stem (Fig 5L-[Fig pone.0318324.g005]M).

## Discussion

Efficient plant transformation is a bottleneck in many research frameworks. Available approaches are often high input and labor intensive, require specialized equipment, or are not established for many plant species. Tissue culture-independent transformation offers a promising approach to circumvent these difficulties, and recent studies have demonstrated that this approach is not only a possible but feasible [15–17,19,20].

Tissue culture-independent transformation of woody species has been previously achieved in citrus, by direct injection of meristems following micro-wounding [27], or incubation of excised seedling tips in an *Agrobacterium* solution [28]. Similarly, incubating excised *Jatropha curcas* seedling tips in *Agrobacterium* following needle pricking, was able to drive regeneration of transgenic shoots [29], as was a similar method used in *Corchorus olitorius* [18]. Asides from successful transformation, tissue culture-independent regeneration was achieved, in pomegranate [30], *Ziziphus acidojujuba* [31] *Ziziphus jujuba* [32] and tomato [33].

For these reasons, we attempted to adopt an existing transformation system to three woody species – tree tobacco, cotton and coffee. We achieved transgenic shoots in tree tobacco and transgenic calli in cotton, but coffee proved to be the least amenable to the protocols that we used. However, despite the successes with tree tobacco and cotton, we were not able to reach transgenic seeds, or even transgenic non-callus tissue in cotton.

Reasons for our lack of success could be technical, of course, and the systems we used may need further calibration. However, our attempts with multiple plant species, bacterial strains, plasmid systems, promoters and delivery methods indicate that there are fundamental issues with the use of growth regulators for tissue culture-independent transformation in the format which we used. *WUS* is a transcription factor that is responsible for specification of the stem cells in the center of the shoot apical meristem [34]. This master regulator has multiple feedback loops [35,36], and both increasing *WUS* expression [37] and reducing it [38] leads to severe growth defects. Reducing *WUS* expression can lead to differentiation of the shoot apical meristem and depletion of its stem cell pool. Conversely, increasing *WUS* expression increases the meristem size, causes fasciation, and will lead to plants that are incapable of producing seeds. *WUS* overexpression may also lead to lethality [39], which is one of the reasons why *WUS* overexpression studies often use inducible promoters [26,40,41]. Using *WUS2* from maize has potential to be less damaging than the Arabidopsis *WUS*. However, as we saw in this study, when we do manage to achieve proper transformation events, these often lead to extremely severe growth defects, which even prior to reaching their final size, could be evaluated as unable to reach seed production. Furthermore, even when plant organs aren’t severely misshapen, growing slightly slower often leads to normal-growing branches further inhibiting the transgenic shoot’s growth, either through being a stronger sink for resources or by preventing light from reaching transgenic shoots. We had to expend great effort pruning plants to favor transformed branches and keep these visible and alive.

In addition to these somewhat technical issues, there is a more fundamental issue with WUS’s mode of action. WUS does not act in the cells where the gene is expressed [42]. Even when expressing *WUS* ectopically, two distinct regions are formed – a *WUS*-expressing organizing center and a *WUS* expression free *CLV3*-expressing meristematic region [26]. If this is also the case when expressing *ZmWUS2*, which was originally characterized based on its similarity to Arabidopsis *WUS* [43], even shoots that are formed as a result of the transformation may be non-transgenic. This can also explain the phenotype of the mature tree tobacco transformants, which have shoot-like structures formed from calli, where the “leaves” are not pink, but the core is. This pink core could correspond to the *WUS*-expressing organizing center.

One approach to achieving tissue culture-independent transformation would be to use tissue-specific or inducible promoters. For instance, targeting *WUS* expression to the cambium using the poplar PXY promoter [44] could lead to the formation of ectopic shoots without the formation of calli that will inhibit the further growth of the ectopically formed shoots. Another option, is to use inducible promoters, as has already been achieved successfully using a WUS-GR system [40]. In this way, following the formation of calli or ectopic shoots, the induction can be stopped, thus leading to differentiation of the callus to shoots.

These suggestions may aid in reaching the production of transgenic seeds in tree tobacco and cotton, however, in coffee the issue is different. This plant was not as susceptible to the transformation system we used. Since coffee has been shown to respond to Arabidopsis *WUS*, forming somatic embryos [45], we suggest using this same *WUS*, or the endogenous coffee *WUS* in a similar tissue-specific or inducible manner.

Transferring of the transformation from the somewhat rigid Goldengate system to the extremely flexible Goldenbraid system facilitates attempts to transform many additional plant species while fine-tuning the system. Expression intensity and location can be fine-tuned through promoter replacement. Different reporter genes can be used, as not all plants will produce betalains upon *RUBY* expression. Species-specific endogenous genes and promoters can be used when the more general genes fail to induce the wanted reactions.

Tissue culture-independent transformation offers an enticing option for generating transgenic or genome-edited plants. Tissue culture-mediated transformation is a long, expensive and labor-intensive process, and skipping this stage sounds like a major improvement of the transformation procedure. However, it is important to realize, that unlike virus-mediated germline editing using CRISPR/Cas9 [46], which saves months of work [47], tissue culture-independent transformation can take as much time as tissue culture-mediated transformation. Furthermore, tissue culture-independent transformation requires a strong visible reporter. When performing tissue culture-mediated transformation, usually antibiotic resistance genes serve as reporters of successful transformation events. These selections are quite robust, but even so, there may be false positive results. In a tissue culture-independent transformation system, when we express a gene and want neither the growth regulator nor the reporter (be it betalain production or *PDS* knock out) to end up in our final transgene, this leads to another problem – how to robustly select a transformed tissue? In our case, many shoot tissues originating in pink calli or harboring growth defects that indicate the presence of the growth regulators, turned out to be non-transgenic. Therefore, when transforming plants that have an established protocol that is not exceedingly time consuming (*e.g*., tomato and tobacco species), the traditional tissue culture-mediated transformation protocols might be a better option. By contrast, tissue culture-independent transformation could be extremely beneficial if it were to replace protocols currently used for plant species where the transformation process is exceedingly long (*e.g*., coffee), species that are difficult to transform (*e.g*., cannabis), or even for developing transformation systems for previously untransformed plants. Developing such a protocol in tissue culture requires fine tuning of *Agrobacterium* strains, hormone concentrations and growth conditions. If a toolkit were to be developed containing Goldenbraid parts of different promoters, including inducible and tissue specific promoters, growth regulators from several plant families, and a range of reporter genes, examining the response of a plant to several of these may be a relatively simple and swift process.

## Supporting information

S1 Fig Goldenbraid plasmid generation workflow. (TIF)

S2 Fig PUPD-*NOS* promoter + PUPD2-*ZmWUS2* + PUPD-*NOS* terminator -> α1-*PNOS*::*WUS*-*TNOS.* (TIF)

S3 Fig PUPD-*35S* promoter + PUPD2-*AtIPT* + PUPD-*35S* terminator -> α2-*P35S*::*IPT*-*T35S.* (TIF)

S4 Fig PUPD2-*e35S* promoter + PUPD2-*ZmWUS2* + PUPD-*35S* terminator -> α1-*e35S*::*WUS*-*T35S.* (TIF)

S5 Fig PUPD-*AtUB10* promoter + PUPD2-*AtIPT* + PUPD2-*SlUB10* terminator -> α2-*AtUB10*::*IPT*-*SlUB10.* (TIF)

S6 Fig PUPD2-*YLCV* promoter + PUPD2-*RUBY* + PUPD2-*NOS* terminator -> α2-*YLCV*::*RUBY*-*TNOS.* (TIF)

S7 Figα1-*PNOS*::*WUS*-*TNOS+*α2-*P35S*::*IPT*::*T35S* -> Ω2-*NOS*::*WUS*+*35S*::*IPT.*(TIF)

S8 Figα1-*e35S*::*WUS*-*T35S+*α2-*AtUB10*::*IPT*::*SlUB10* -> Ω2-*e35S*::*WUS*+ *UB10*::*IPT.*(TIF)

S9 Figα1-SF (stuffer)*+*α2-*YLCV::RUBY-TNOS* -> Ω1-*YLCV*::*RUBY.*(TIF)

S10 FigΩ1-*YLCV::RUBY+* Ω2-*NOS::WUS+35S::IPT* -> α1-*YLCV*::*RUBY + NOS::WUS +35S::IPT* (GB-*NOS*::*WUS*.)(TIF)

S11 FigΩ1-*YLCV::RUBY+* Ω2-*e35S::WUS+UB10::IPT* -> α1-*YLCV*::*RUBY + e35S::WUS +UB10::IPT* (GB-*e35S*::*WUS*).(TIF)

S12 Fig A flowchart of the different plasmid systems, constructs and bacteria used in each of the four species examined. GG-OE – a Goldengate overexpression plasmid, including *NOS*::*WUS*, *35S*::*IPT* and *35S*::*GFP*. GG-Cas9 – a Goldengate plasmid including *NOS*::*WUS*, *35S*::*IPT, Cas9* and U6::*NbPDS-guideRNA. GB-NOS::WUS* – a Goldenbraid plasmid including *NOS*::*WUS*, *35S*::*IPT* and *YLCV*::*RUBY*. GB-*e35S*::*WUS* – a Goldenbraid plasmid including an enhanced 35S promoter driving *WUS* expression, *UB10*::*IPT* and *YLCV*::*RUBY*.
(TIF)

S13 Fig PCR reactions targeting the *ZmWUS* gene performed for cotton leaves and calli in plants that were infiltrated with GB- *NOS::WUS* as well as GB- *NOS::WUS* +GFP. Only calli samples were positive for the presence of *ZmWUS*, whereas leaves, even when originating in a pink callus were not positive for the gene. Cotton variants and constructs used are indicated above corresponding lanes. Bacterial strains used are indicated below the gel, as are the tissues profiled. P16 – Delta Pine 16 cotton.
(TIF)

S1 Table A list of primers used for domestication of Goldenbraid parts. (DOCX)
