# Supplementary figures and images for: Success and limitations in adaptation of Fast-TrACC tissue culture-independent transformation in coffee, cotton, and tree tobacco
Source: PLoS One. 2025 May 15;20(5):e0318324. doi: 10.1371/journal.pone.0318324 (PMC12080836; doi:10.1371/journal.pone.0318324)

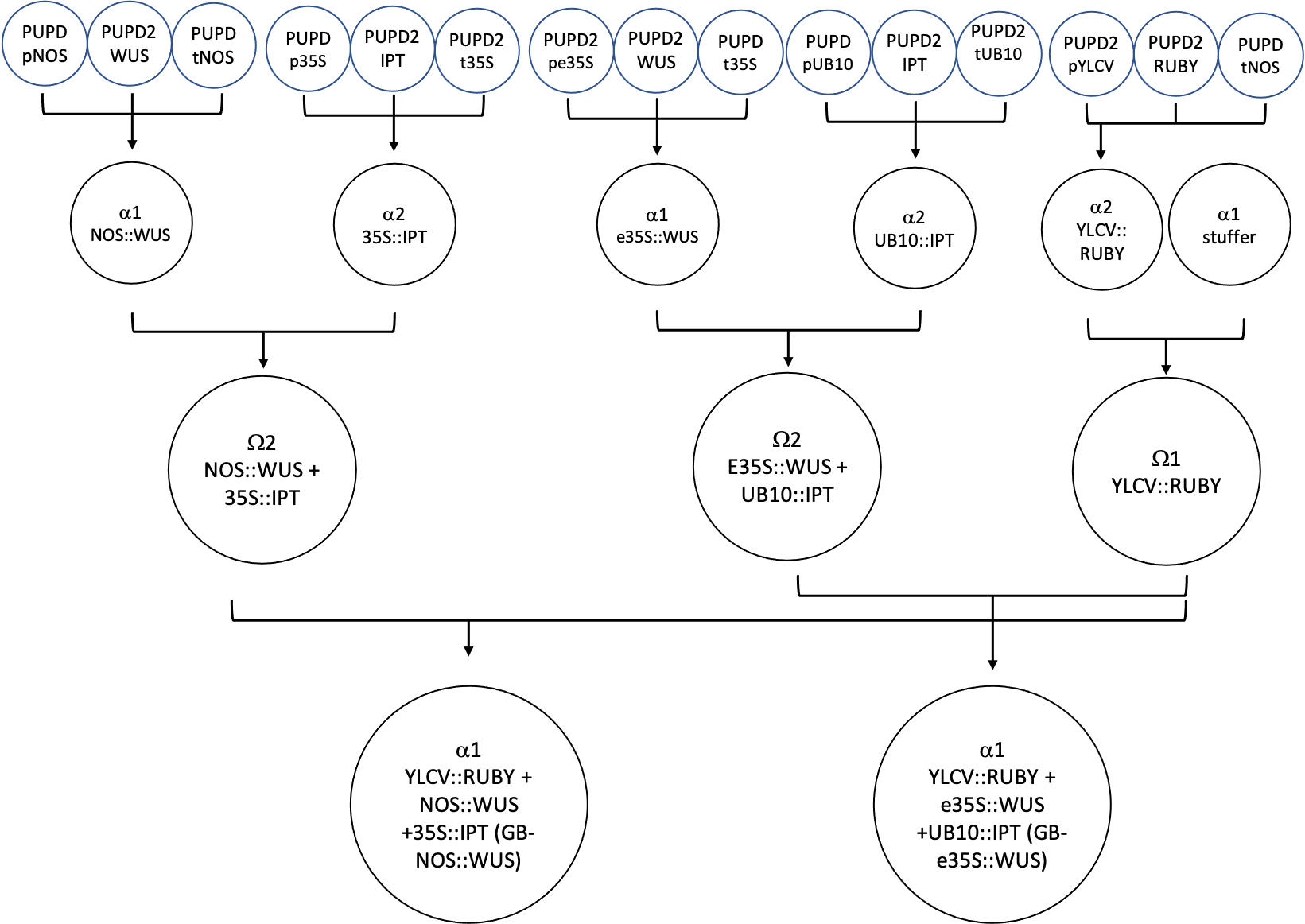

Supplement: S1 Fig — (TIF) [file pone.0318324.s001.tif]

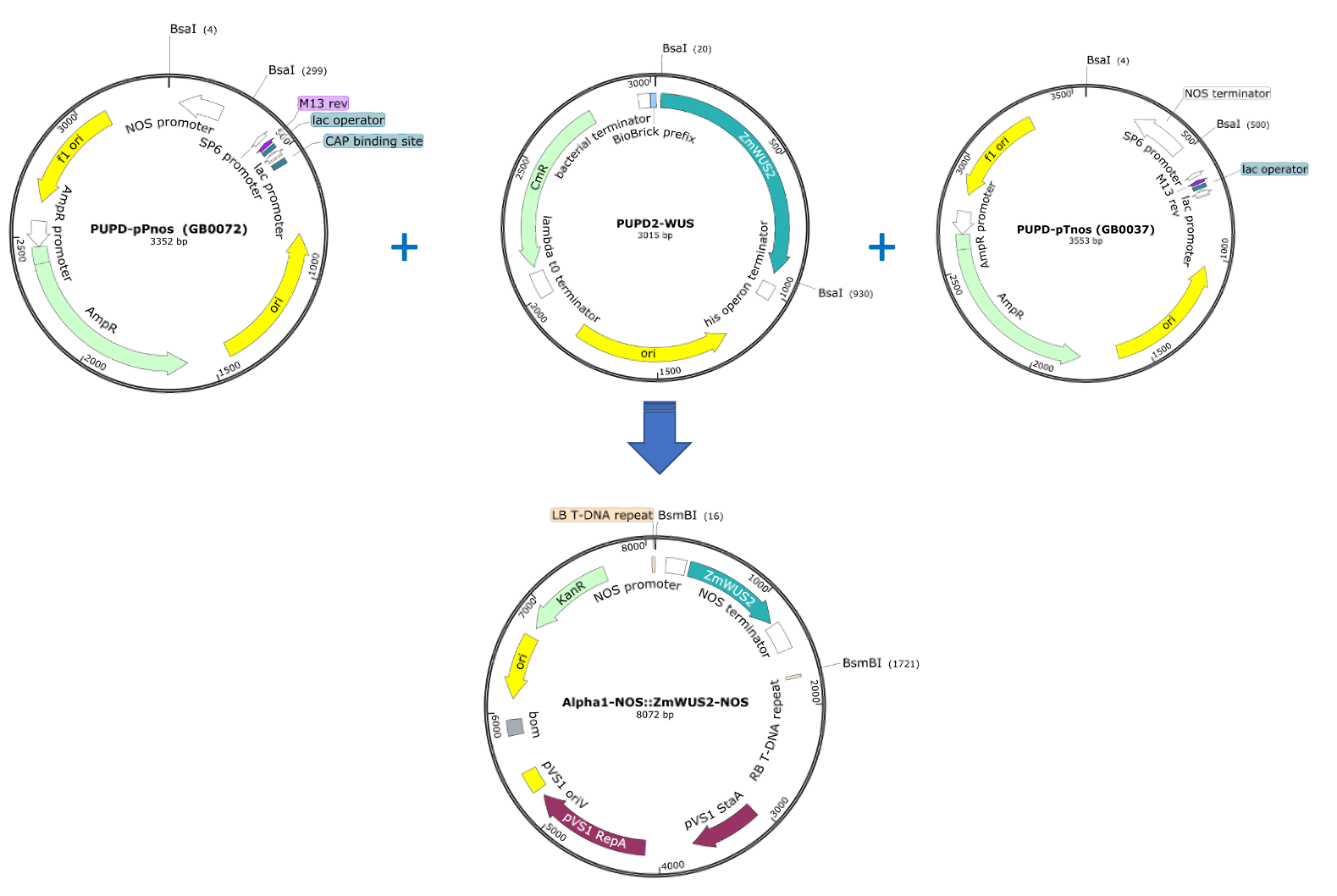

Supplement: S2 Fig — (TIF) [file pone.0318324.s002.tif]

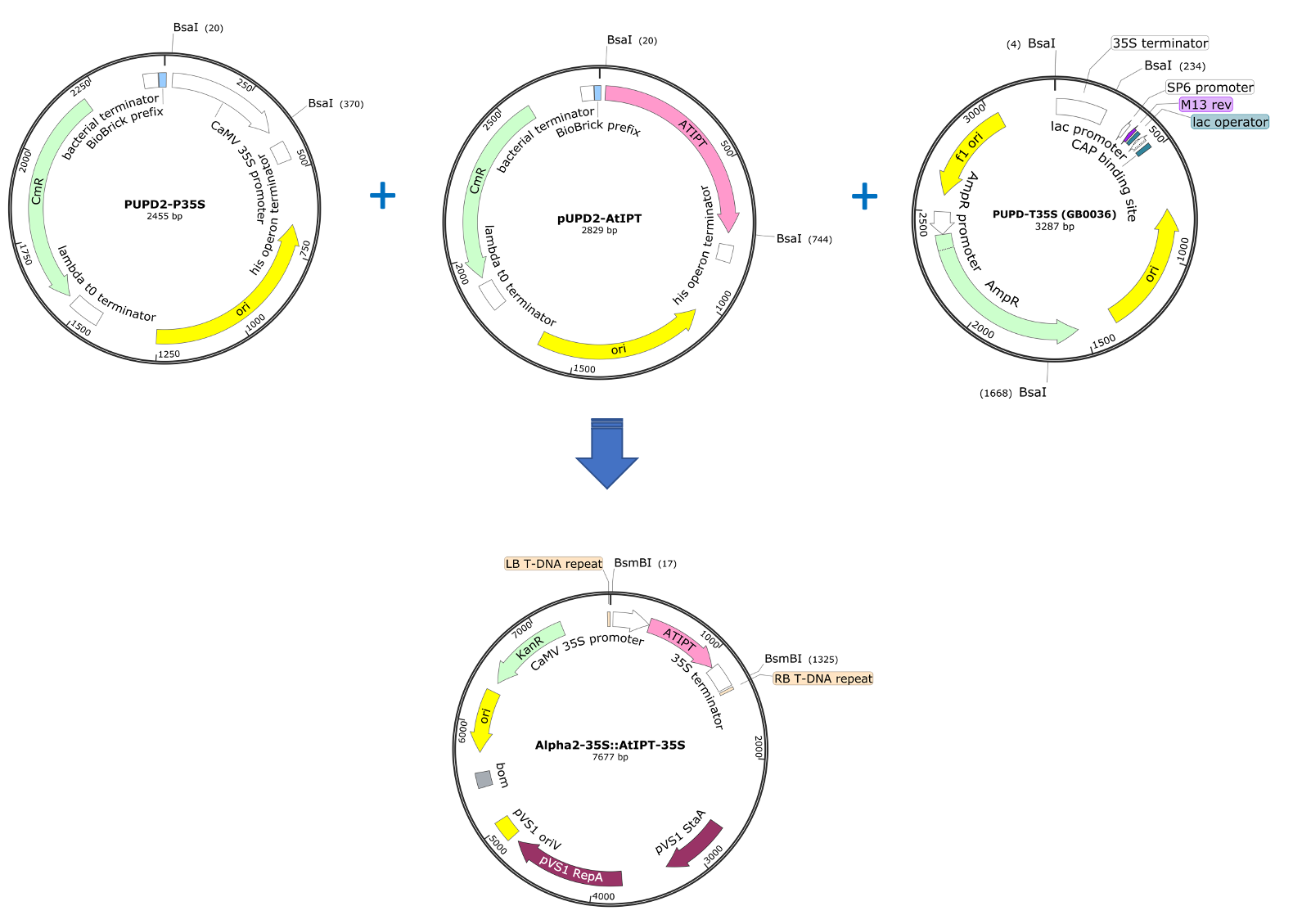

Supplement: S3 Fig — (TIF) [file pone.0318324.s003.tif]

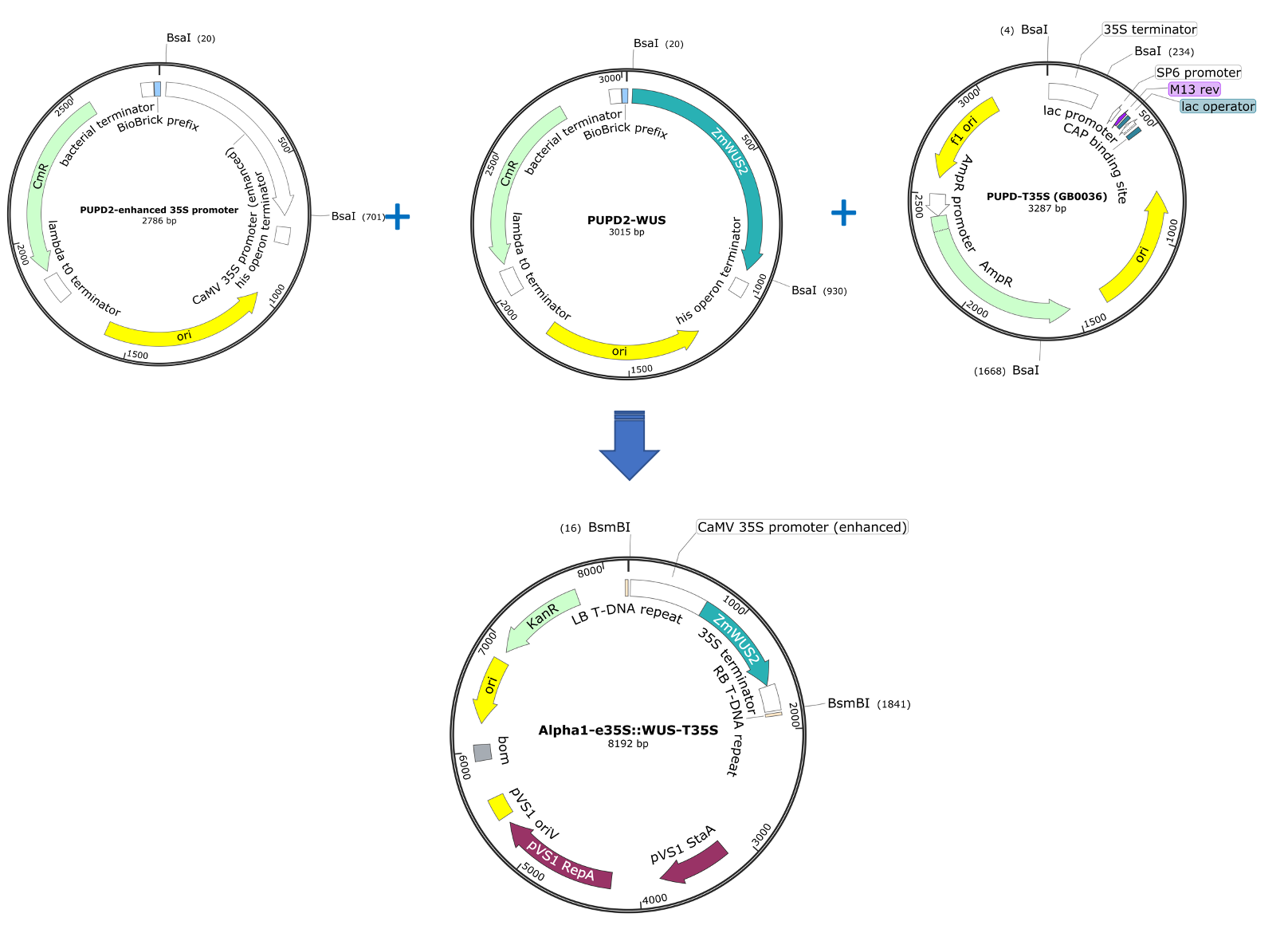

Supplement: S4 Fig — (TIF) [file pone.0318324.s004.tif]

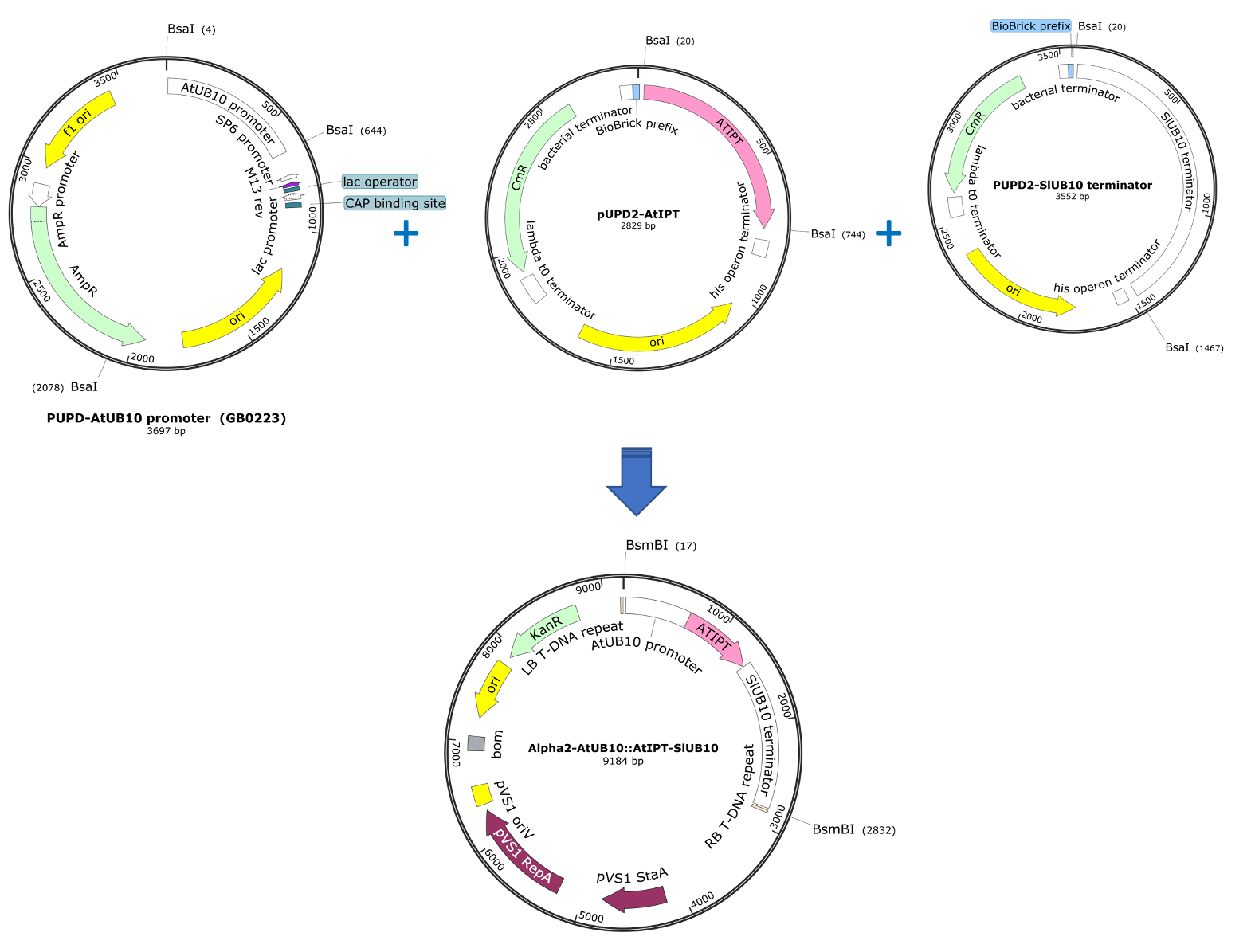

Supplement: S5 Fig — (TIF) [file pone.0318324.s005.tif]

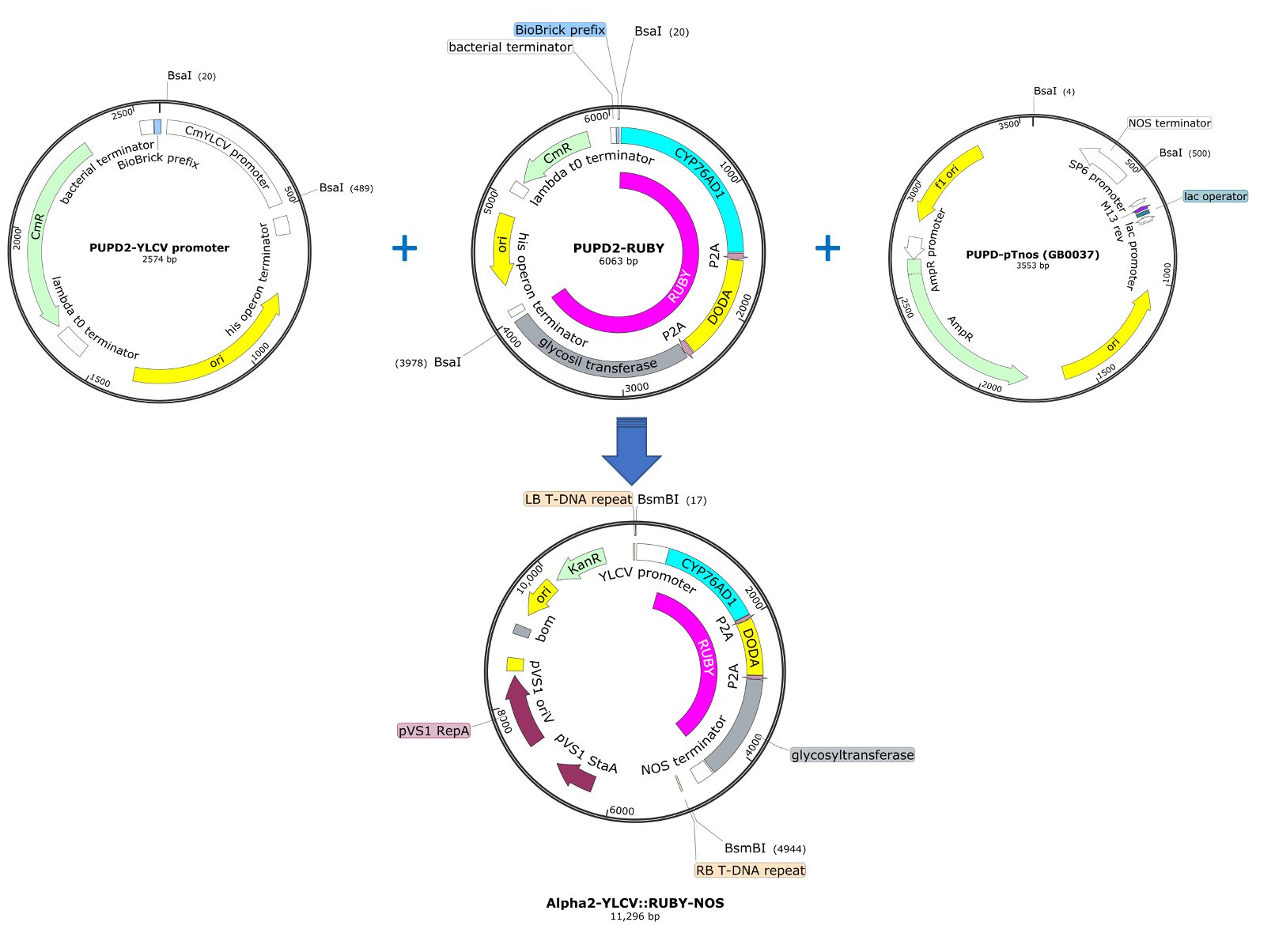

Supplement: S6 Fig — (TIF) [file pone.0318324.s006.tif]

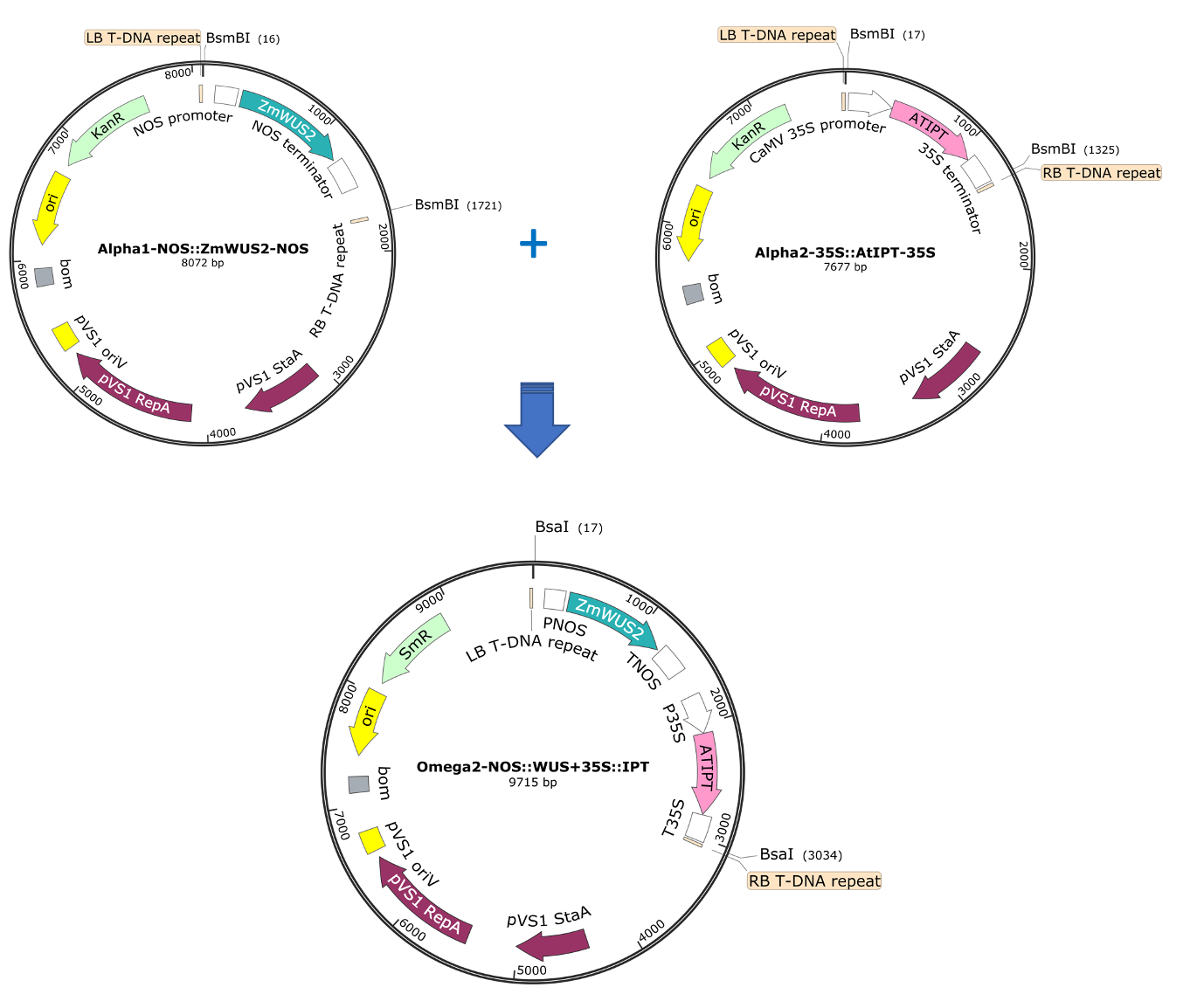

Supplement: S7 Fig — (TIF) [file pone.0318324.s007.tif]

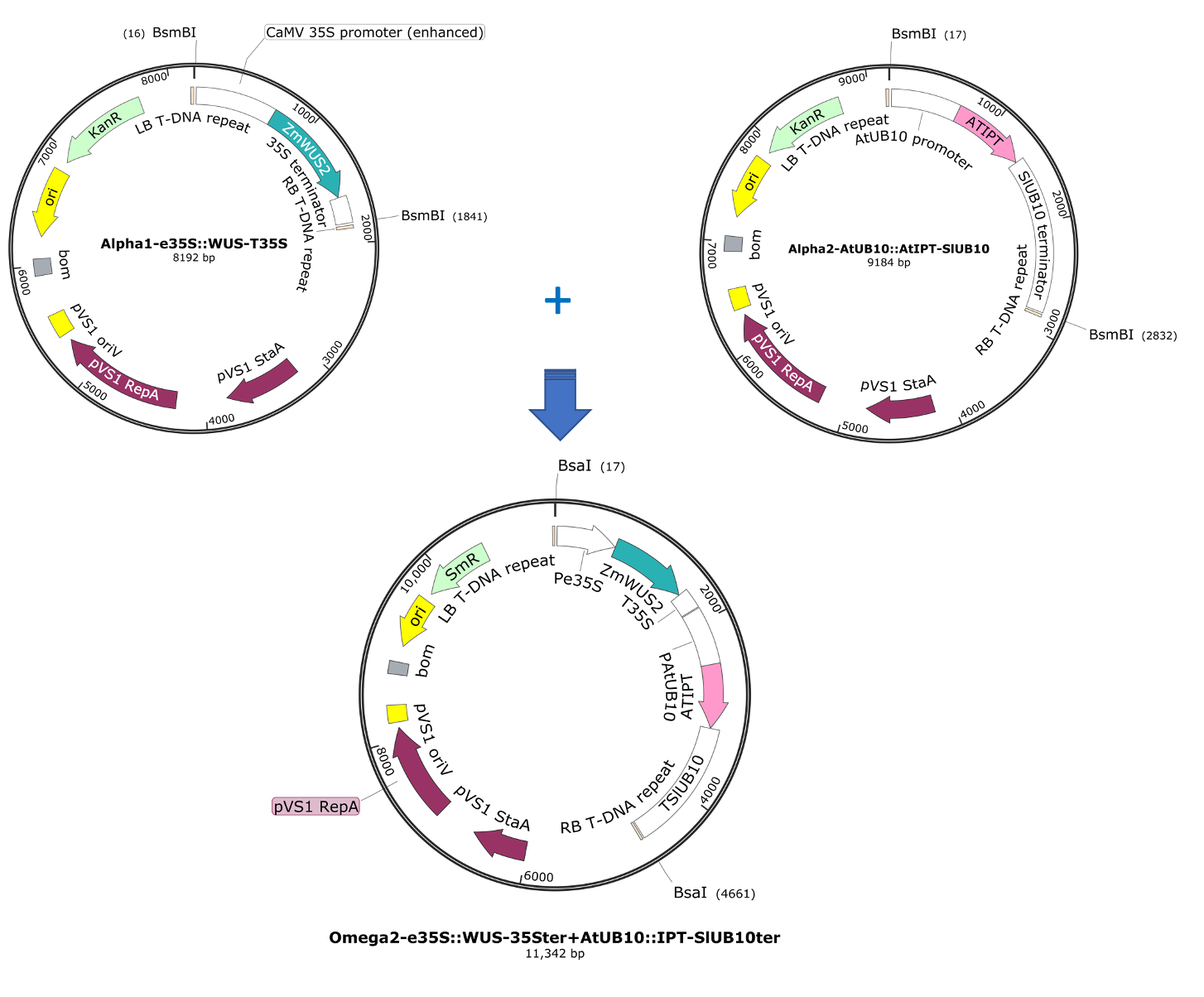

Supplement: S8 Fig — (TIF) [file pone.0318324.s008.tif]

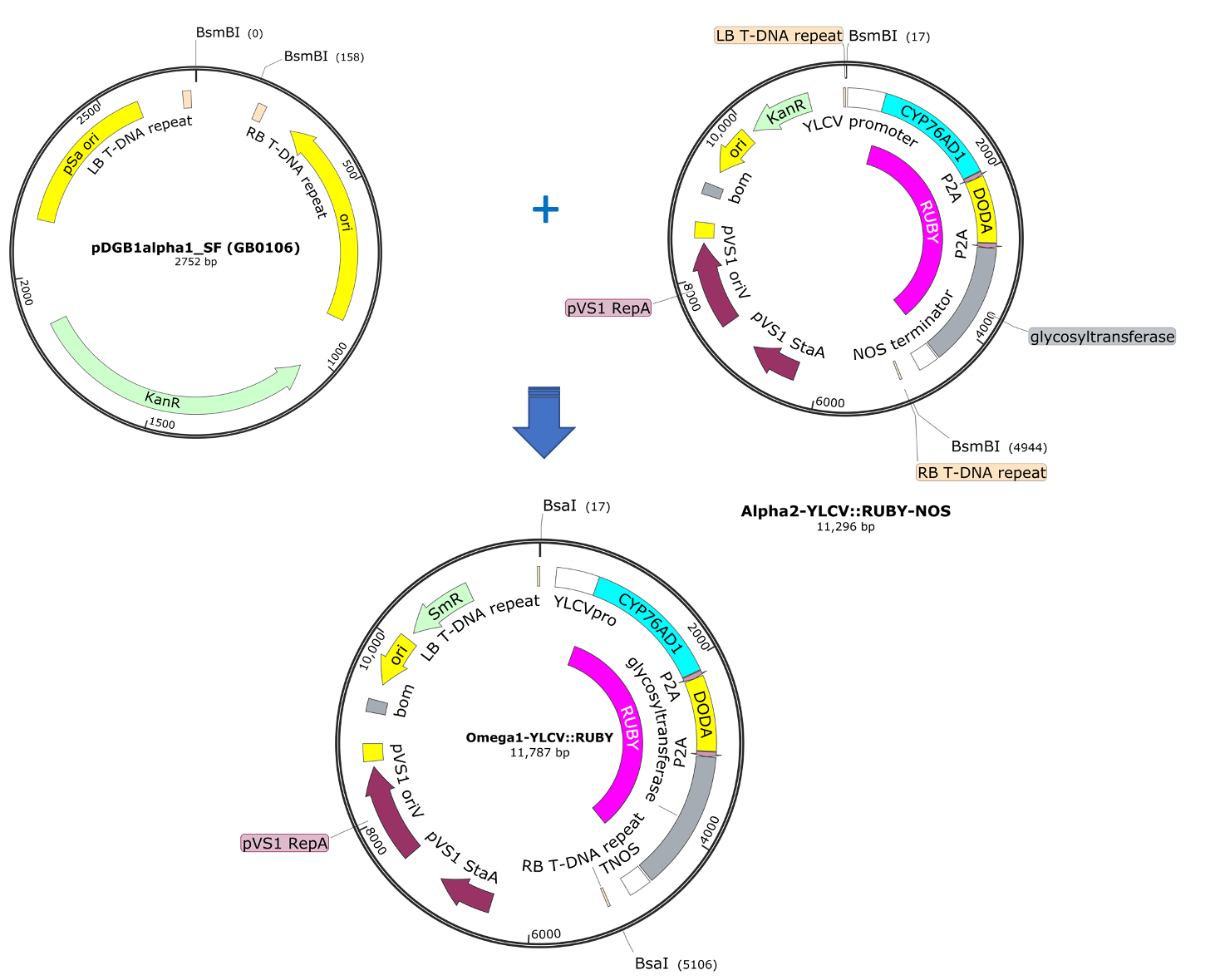

Supplement: S9 Fig — (TIF) [file pone.0318324.s009.tif]

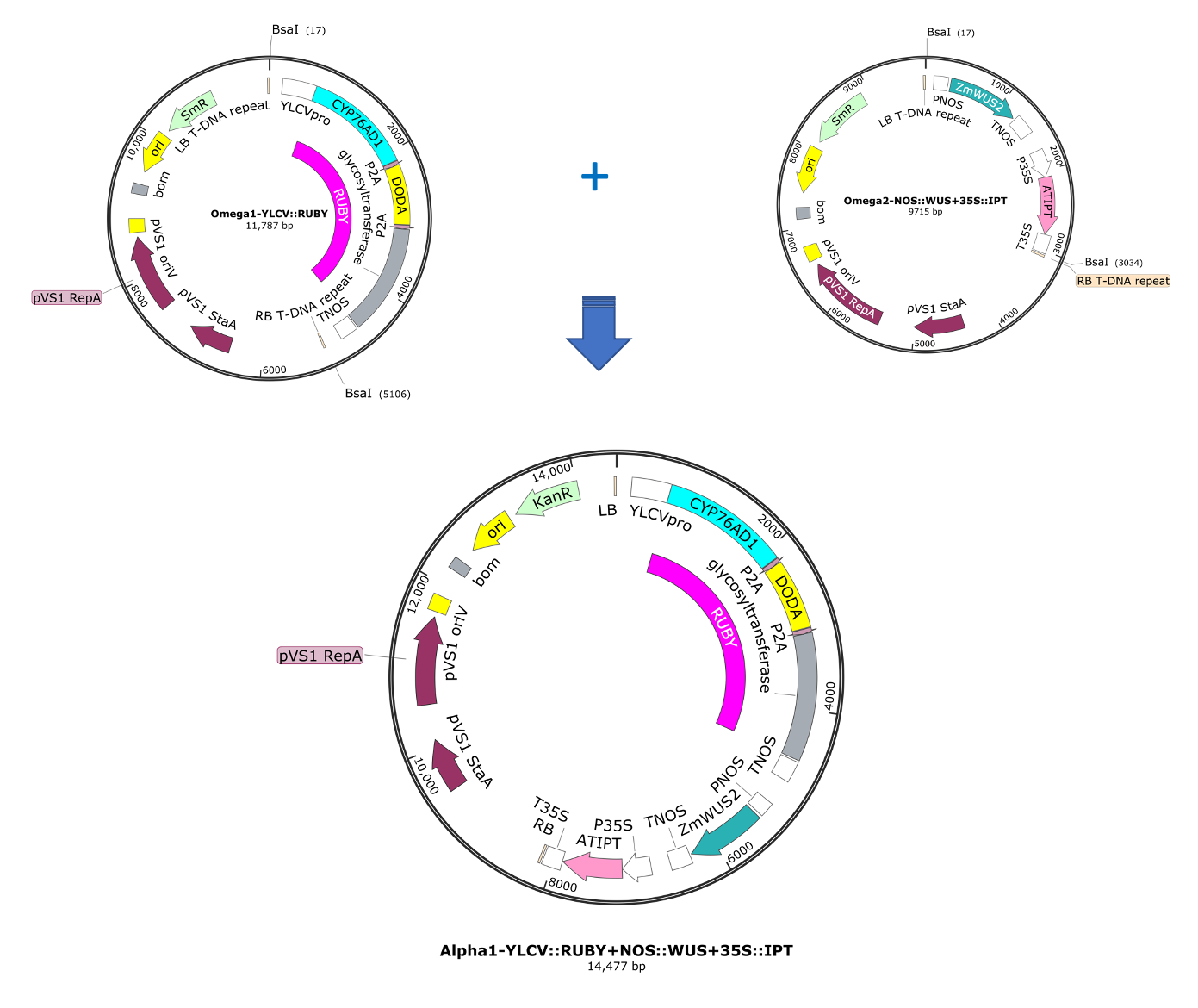

Supplement: S10 Fig — (TIF) [file pone.0318324.s010.tif]

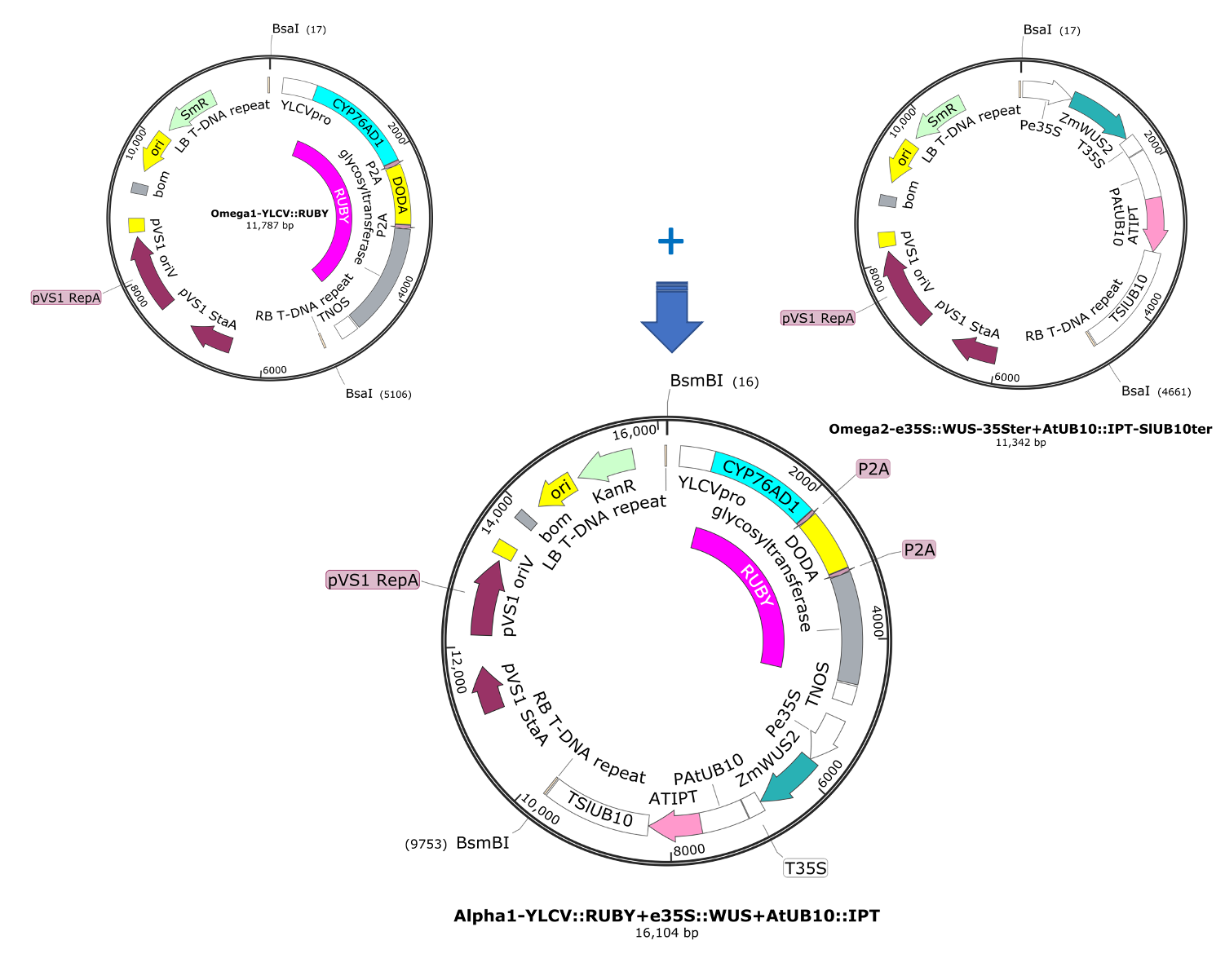

Supplement: S11 Fig — (TIF) [file pone.0318324.s011.tif]

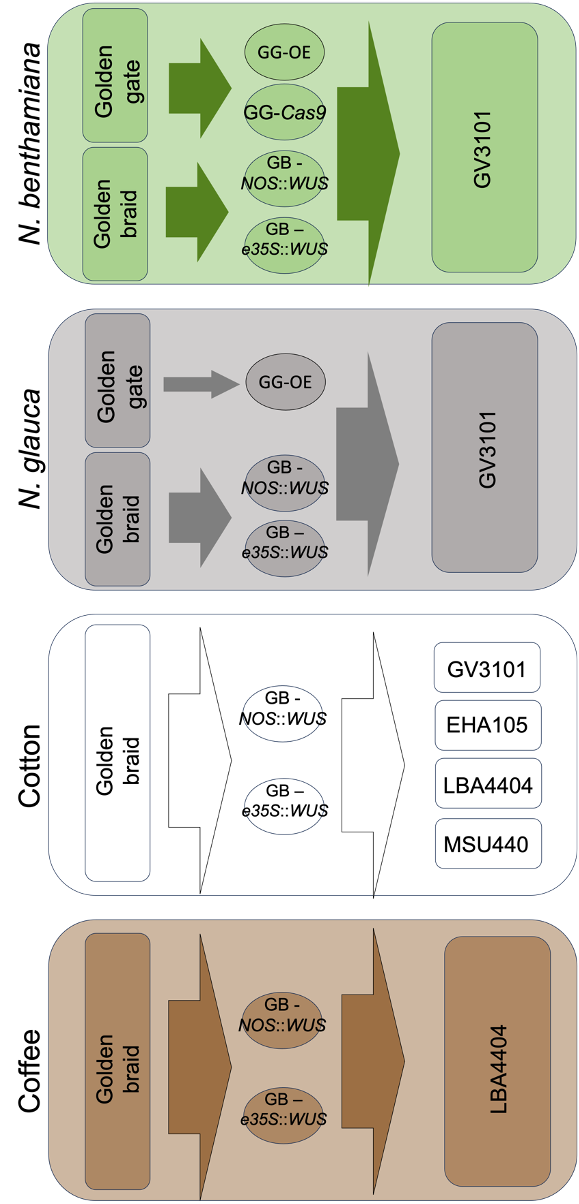

Supplement: S12 Fig — GG-OE – a Goldengate overexpression plasmid, including NOS::WUS, 35S::IPT and 35S::GFP. GG-Cas9 – a Goldengate plasmid including NOS::WUS, 35S::IPT, Cas9 and U6::NbPDS-guideRNA. GB-NOS::WUS – a Goldenbraid plasmid including NOS::WUS, 35S::IPT and YLCV::RUBY. GB-e35S::WUS – a Goldenbraid plasmid including an enhanced 35S promoter driving WUS expression, UB10::IPT and YLCV::RUBY. (TIF) [file pone.0318324.s012.tif]

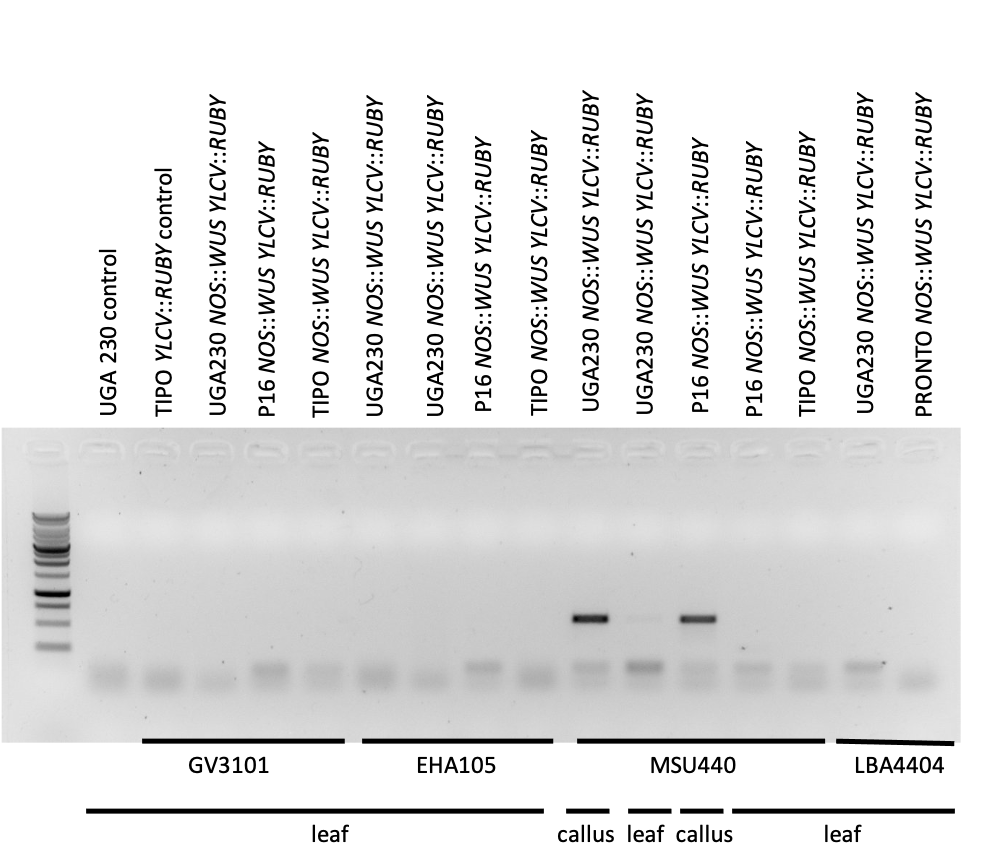

Supplement: S13 Fig — Only calli samples were positive for the presence of ZmWUS, whereas leaves, even when originating in a pink callus were not positive for the gene. Cotton variants and constructs used are indicated above corresponding lanes. Bacterial strains used are indicated below the gel, as are the tissues profiled. P16 – Delta Pine 16 cotton. (TIF) [file pone.0318324.s013.tif]
