# Supplementary material for: Success and limitations in adaptation of Fast-TrACC tissue culture-independent transformation in coffee, cotton, and tree tobacco
Source: PLoS One. 2025 May 15;20(5):e0318324. doi: 10.1371/journal.pone.0318324 (PMC12080836; doi:10.1371/journal.pone.0318324)
Supplement: S1 Table — (DOCX) [file pone.0318324.s014.docx]

| Amplification target | Primer name | orientation | sequence |
| --- | --- | --- | --- |
| *WUS* domestication to pUPD2 | ZmWUS2_domF | F | GCGCCGTCTCGCTCGAATGGCGGCCAATGCGGGCGG |
|  | ZmWUS2_domR | R | GCGCCGTCTCGCTCAAAGCTCACATACTCCCTGCAGCAG |
| *IPT* domestication to pUPD2 | AtIPT_DOM_F | F | GCGCCGTCTCGCTCGAATGGATCTGCGTCTAATTTTCG |
|  | AtIPT_DOM_R | R | GCGCCGTCTCGCTCAAAGCCTAGCACATTCCGAACGGTG |
| *RUBY* domestication to pUPD2 | RUBY_domF | F | GCGCCGTCTCGCTCGAATGGATCATGCGACCCTCGC |
|  | RUBY_domR | R | GCGCCGTCTCGCTCAAAGCTCACTATCACTGGAGGCTTG |
| *PPDK* promoter domestication to pUPD2 | enhanced_35SP_domF | F | GCGCCGTCTCGCTCGGGAGTGAGACTTTTCAACAAAGGGTAAT |
|  | enhanced_35SP_domR | R | GCGCCGTCTCGCTCACATTTCAGCGTGTCCTCTCCAAAT |
| *E35S* promoter domestication to pUPD2 | enhanced_35SP_domF | F | GCGCCGTCTCGCTCGGGAGTGAGACTTTTCAACAAAGGGTAAT |
|  | enhanced_35SP_domR | R | GCGCCGTCTCGCTCACATTTCAGCGTGTCCTCTCCAAAT |
| *YLCV* promoter domestication to pUPD2 | YLCVpro_domF | F | GCGCCGTCTCGCTCGGGAGTGGCAGACATACTGTCCCAC |
|  | YLCVpro_domR | R | GCGCCGTCTCGCTCACATTAAGCTTAGCTCTTACCTGTTTTC |
| GG *35S* promoter domestication to pUPD2 | enhanced_35SP_domF | F | GCGCCGTCTCGCTCGGGAGTGAGACTTTTCAACAAAGGGTAAT |
|  | Voytas_35Spro_domR | R | GCGCCGTCTCGCTCACATTTGTTCTCTCCAAATGAAATGAACT |
| *ZmWUS2* identification | WUS2_117F | F | CAGGATGCTGAAGGAGCTGT |
|  | WUS2_644R | R | GTCGGGAAGAGAGGGAGAGT |
| *RUBY* identification | ruby_detection_F | F | GCCGAGACAGACCAAAAGAG |
|  | ruby_detection_R | R | AGTGCTCTGGAAATGGATGG |
